# Rapid Method of Identifying Typhoid Baccilli

**Published:** 1915-11

**Authors:** 


**Rapid Method of Identifying Typhoid Baccilli.** Loewenfeld, Munch. Med. Woch., No. 13, 1915, to 10 C. C. of nutrient broth, add 1 drop of highly potent agglutinating serum (titer 1:8000)

Add several loops from Conradi, blood-bile culture tubes. Incubate for 6 hours. The agglutinated bacilli form a fine floculent precipitate.

